# The protective effect of resveratrol on lupus nephritis mice by up-regulating Sirt1

**DOI:** 10.22038/ijbms.2025.90231.19451

**Published:** 2026

**Authors:** Yu Zhao, Yun Cai, Jie Chen, Xu-yan Yang

**Affiliations:** 1 Department of Rheumatology, The Second Affiliated Hospital Zhejiang University School of Medicine, Hangzhou, Zhejiang Province, P.R. China; 2 Department of Nephrology, Ningbo Yinzhou No.2 Hospital, Ningbo, Zhejiang Province, P.R. China

**Keywords:** Apoptosis, Lupus nephritis, Oxidative stress, Resveratrol, Sirt1

## Abstract

**Objective(s):**

To investigate the therapeutic effect and protective mechanism of resveratrol (Res) on mice with lupus nephritis (LN).

**Materials and Methods:**

We used Res to intervene in MRL/lpr mice and employed biochemical techniques to assess kidney function, inflammation levels, and oxidative stress. We also evaluated changes in immune function and used immunohistochemistry to assess Sirt1 protein and mRNA expression.

**Results:**

Res improved the kidney function, alleviated proteinuria, and renal pathological damage in MRL/lpr mice. Res also ameliorated spleen weight and spleen index, and inhibited urinary protein/creatinine levels. Moreover, Res inhibited the expression of circulating inflammatory factors, oxidative stress levels, and kidney cell apoptosis in MRL/lpr mice. Res effectively promoted Sirt1 protein and mRNA expression in the kidneys of MRL/lpr mice.

**Conclusion:**

Res has a protective effect on MRL/lpr mice, and its mechanism may involve activation of the Sirt1 signaling pathway.

## Introduction

Systemic lupus erythematosus (SLE) is a multisystem, chronic, diffuse connective tissue disease of unclear etiology that can involve multiple organs (1). Lupus nephritis (LN) mainly affects women of childbearing age from 20 to 50 years old. The male-to-female ratio stands at 1:9, with notable ethnic variations also observed. The incidence rate of black people is the highest, followed by the Han population in China (2). According to the survey, LN ranks among the SLE complications with the highest mortality rates, accounting for 25% (3). The pathogenesis of LN mainly involves immune system disorders, production of autoantibodies, and release of tissue-damaging substances (4). Therefore, the treatment of LN primarily relies on a combination of immunosuppressants and hormones. However, 30% of immunosuppressive therapy patients eventually progress to renal failure, and high-dose immunosuppressive agents can cause irreversible damage to the kidneys (5). 

*Polygonum cuspidatum* is a traditional Chinese medicinal (TCM) herb in China, among which resveratrol (Res) is the main pharmacologically active ingredient, which has anti-inflammatory, anti-fatigue, anti-aging, oxidative stress relief, cardiovascular protection, and other effects (6). Experimental results showed that Res has a good protective effect on tissues damaged by hypoxia (7). Moreover, Res has been widely used in clinical practice to treat acute altitude sickness and diseases caused by chronic hypoxia, suggesting broad prospects for its application in related diseases (8-10). For LN, which urgently needs to expand treatment options, Res has also entered the research scope due to its practical anti-inflammatory effects. Studies have demonstrated that Res ameliorates the proliferation of glomerular mesangial cells and the accumulation of extracellular matrix induced by high-glucose stimulation by inhibiting NLRP3 activation (11). Res also plays a protective role in diabetic kidney disease by improving mitochondrial metabolic function and slowing down oxidative stress (12).

Previous studies have indicated that Res exhibits multiple biological activities, including anti-inflammatory and anti-oxidant effects (11, 12). Res reduces inflammation caused by immune complex deposition by inhibiting T cell activation and by blocking autoantibody production by B cells. However, its protective effect on LN remains unclear; there is limited information on whether RES treatment for LN is associated with Sirt1 regulation. Therefore, the current study employed the classic spontaneous lupus mouse model MRL/lpr to investigate the efficacy and mechanism of Res in LN, with the findings expected to provide a theoretical basis for the development of lupus-targeted drugs.

## Materials and Methods

### Experimental animals and grouping

We used a blind method to randomly divide MRL/lpr mice (12 weeks old) into a disease group, a low-dose Res treatment group (50 mg/kg), and a high-dose Res treatment group (100 mg/kg), with 10 mice per group. Additionally, age-matched C57BL/6 mice (n=10) were included as normal controls. The treatment group started taking Res orally at 14 weeks of age and continued to receive it for 7 weeks. Both the disease group and the normal control group mice were given equal amounts of physiological saline. Mice were euthanized at 21 weeks of age, and samples of spleen, urine, blood, and kidney tissues were collected for subsequent assays. 

### H&E staining

Following euthanasia, kidney tissues were harvested and fixed in 4% paraformaldehyde. After dehydration in a graded ethanol series, the tissues were embedded in paraffin and sectioned at 3 μm for H&E staining. Morphological alterations in the kidney tissues were then observed under a light microscope.

### RT-qPCR

After homogenizing the kidney tissue sample, total RNA was extracted according to the Takara kit instructions (Takara Co., Beijing, China). cDNA was synthesized using a reverse transcription kit, and the mRNA expression level of Sirt1 in kidney tissue was detected by RT-qPCR using the SYBR Green method. Sirt1: 3’-AGA TTT TAA GGC TGT TGG TT-5’, 5’-TGC TCC ACC AGC ATT GGG AA-3’; GAPDH: 3’-GAC CAA GTA CGC TGT GAG TA-5’, 5’-ACT CGT ACA TCC GGA CAC AT-3’. Pre-denaturation: 94 ℃ for 10 sec, amplification cycles: 94 ℃ for 10 sec, 58 ℃ for 20 sec, 72 ℃ for 15 sec, a total of 35 cycles, detection after 72 ℃ extension, GAPDH as an internal reference.

### Renal function

We measured 24-hr urinary protein and creatinine in each group of mice using Albuwell M and Creatinine companion kits, following the instructions for each kit.

### Immune function evaluation

The mice were dissected to obtain spleen and thymus weights in milligrams. Following red blood cell lysis, the single-cell suspensions were divided into two aliquots. One part was added with CD80-PE antibody, CD86-TITC antibody, and CD11 APC antibody, and incubated in the dark for half an hour. The proportion of dendritic cells was analyzed by flow cytometry; CD4-PE antibody, IL-4-TITC antibody, and IFN-γ-APC antibody were added, then incubated in the dark for half an hour. Helper T lymphocyte (Th) subtypes were analyzed using flow cytometry.

### Immunohistochemistry

The tissue paraffin blocks were cut into 4 μm-thick sections, followed by dewaxing, transparency, and other steps. The sections were washed with PBS solution, blocked with 3% bovine serum albumin, and diluted with primary and secondary antibodies (1:200 with PBS, Catalog Number: PB0173, Boster Co., Wuhan, China). The samples were incubated at room temperature.

### TUNEL

Paraffin-embedded tissue sections were prepared, thoroughly dewaxed and hydrated, then incubated with proteinase K. Subsequently, they were stained using the TUNEL assay (Sigma Co., Shanghai, China), followed by DAB staining. After blocking endogenous peroxidase (POD) and rinsing with PBS, the sections were mounted and observed under a fluorescence microscope.

### Statistical analysis

GraphPad Prism 8.25 software was used for graph generation and data analysis. Data conforming to a normal distribution were expressed as mean±standard deviation. One-way analysis of variance (ANOVA) was used for comparisons among multiple groups, with Tukey’s test for *post hoc* pairwise comparisons. A *P*-value<0.05 was considered statistically significant.

## Results

### The protective effect of Res on LN mice

**Figure 1:**
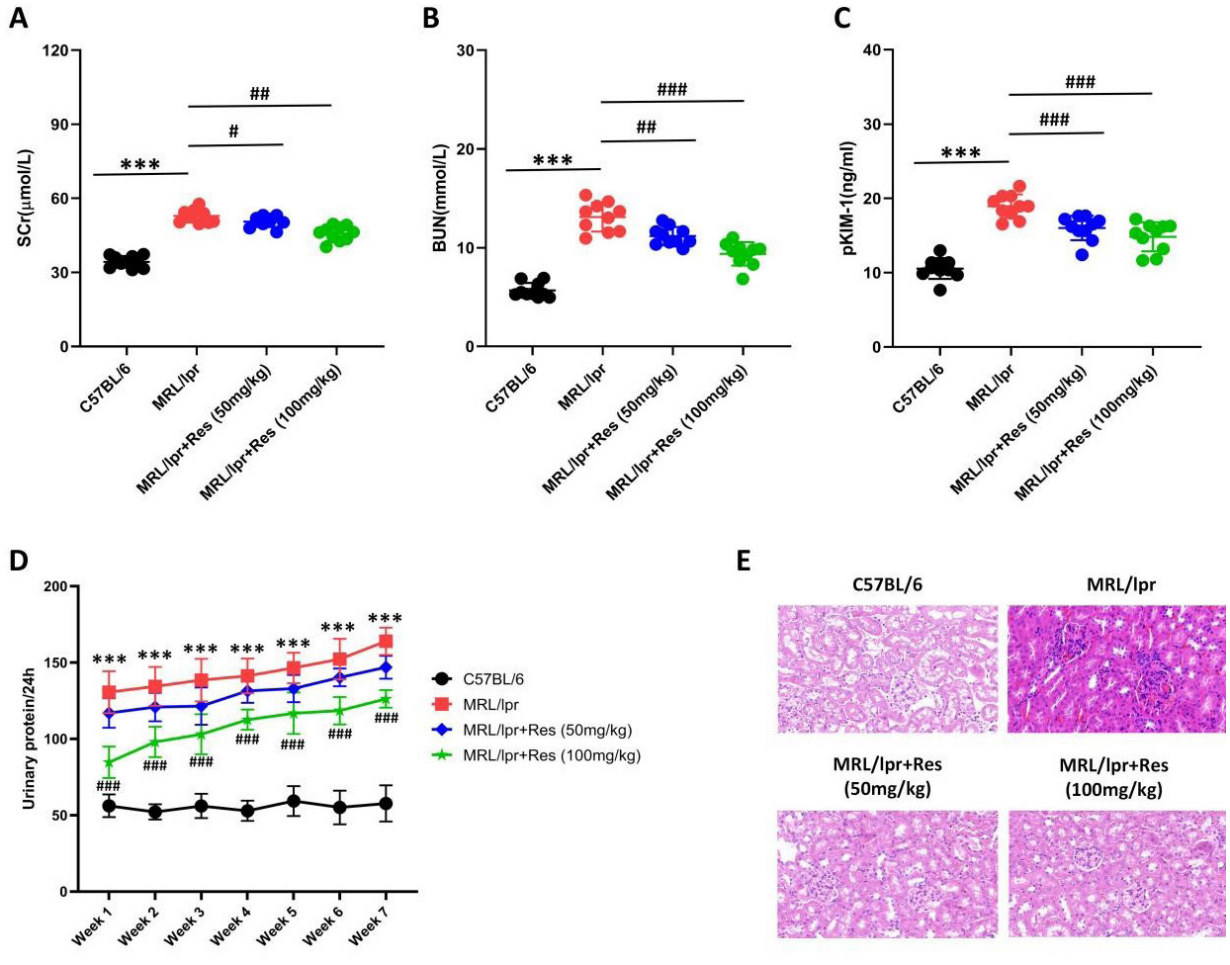
Res exerts a protective effect on LN mice

MRL/lpr mice exhibited significantly elevated renal function indicators, including serum creatinine, blood urea nitrogen, and plasma kidney injury molecule-1 (Figure 1. A-C). These indicators were ameliorated following Res treatment, with the most marked reduction observed at the 100 mg/kg dose (Figure 1. A-C). Concurrently, weekly changes in urinary protein levels were monitored. Urinary protein excretion in MRL/lpr mice increased progressively with age, peaking at 21 weeks (Figure 1D). In age-matched comparisons, Res-treated MRL/lpr mice showed reduced urinary protein excretion, with the most significant reduction at the 100 mg/kg dose (Figure 1D). Pathological evaluation of kidney tissues revealed glomerular enlargement, tubular atrophy, and interstitial fibrosis in MRL/lpr mice; however, Res treatment partially attenuated these pathological manifestations (Figure 1. E). Our findings indicate that Res alleviates renal injury in MRL/lpr mice.

### The effect of Res on the immune function of LN mice

**Figure 2:**
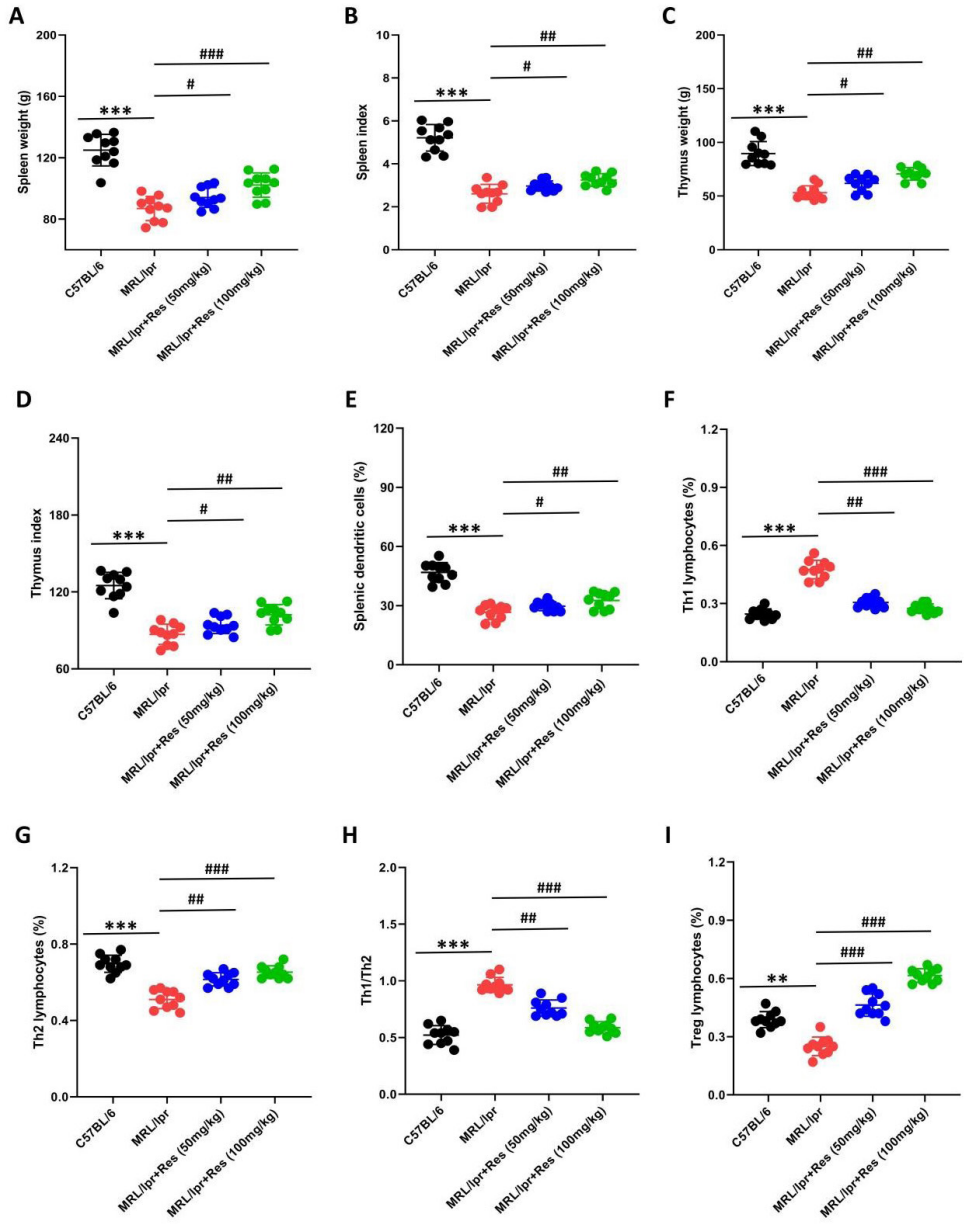
Res restores immune function in LN mice

Immune dysfunction represents a key pathophysiological mechanism underlying LN (13). Accordingly, we investigated the impact of Res on immune function in LN mice. Our findings revealed that spleen weight, spleen index, thymus weight, and thymus index were all reduced in both MRL/lpr mice and MRL/lpr mice treated with Res (Figure 2. A-D). Compared with untreated MRL/lpr mice, those administered two different doses of Res exhibited increased spleen weight and spleen index, and thymus weight and thymus index, with a significant elevation observed in the 100 mg/kg Res group (Figure 2A-D). Furthermore, we assessed splenic dendritic cell and T lymphocyte levels. It was found that Th1 lymphocytes and the Th1/Th2 cell ratio were increased in both MRL/lpr mice and MRL/lpr+Res mice, whereas splenic dendritic cells and the proportions of splenic Th2 and Treg cells were decreased (Figure 2. E-I). Relative to untreated MRL/lpr mice, Res-treated MRL/lpr mice (at two distinct doses) showed reduced Th1 lymphocytes and Th1/Th2 cell ratio, alongside elevated splenic dendritic cells, proportion of splenic Th2 and Treg cells (Figure 2. E-I). These experiments demonstrate that Res can effectively restore immune function in LN mice.

### The effects of Res on cell apoptosis in LN mice

**Figure 3:**
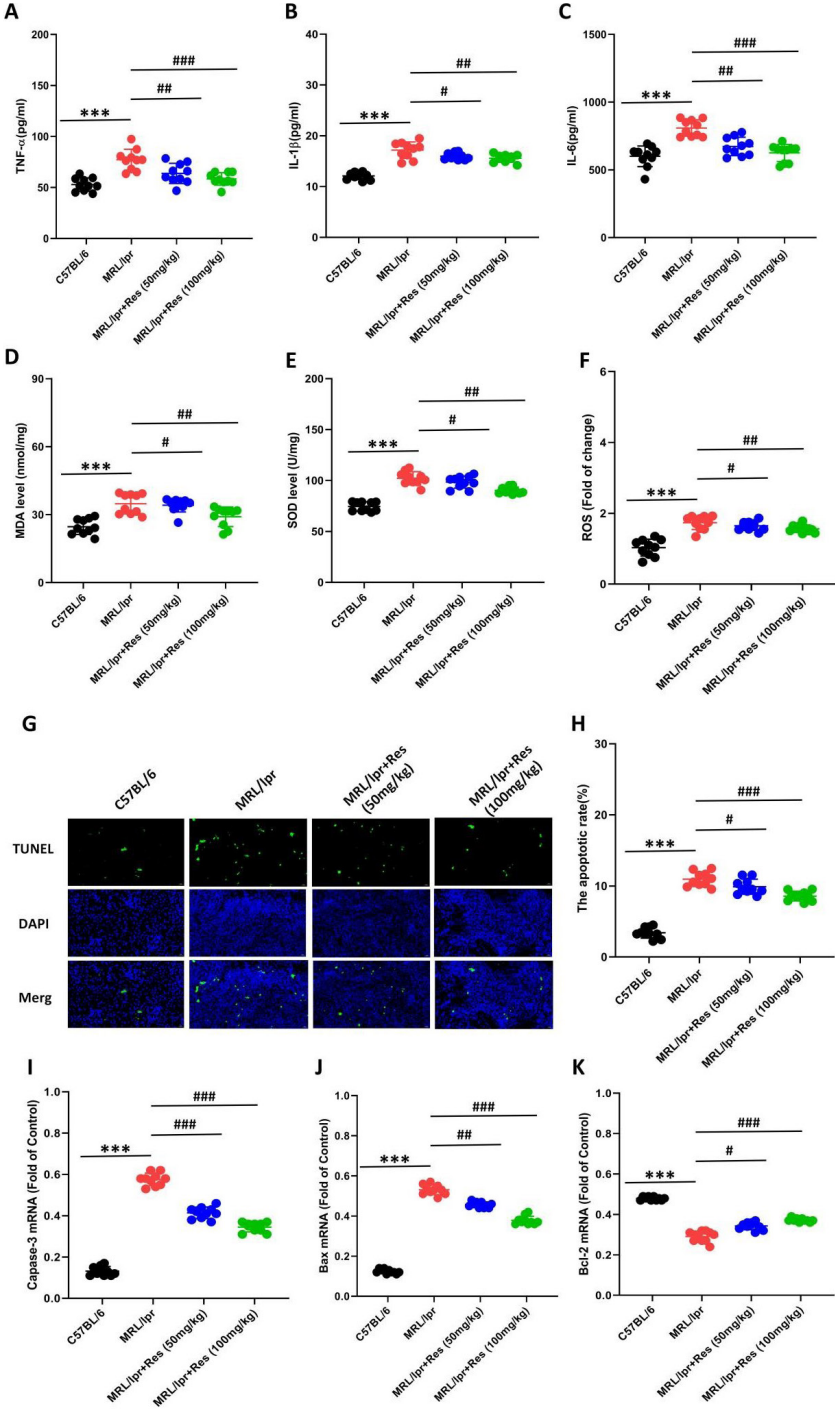
Res has anti-inflammatory, anti-oxidative stress, and anti-apoptotic effects in LN mice

The activation of inflammatory factors and oxidative stress responses constitutes a critical trigger of LN progression (14). Our findings showed that the levels of TNF-α, IL-1β, IL-6, reactive oxygen species (ROS), and malondialdehyde (MDA) were elevated in kidney tissues of both MRL/lpr mice and MRL/lpr mice treated with Res, whereas the level of SOD was reduced (Figure 3. A-F). However, relative to untreated MRL/lpr mice, those treated with Res exhibited decreased levels of TNF-α, IL-1β, IL-6, ROS, and MDA, along with increased SOD levels (Figure 3. A-F). Additionally, we assessed renal cell apoptosis and found that Res significantly reduced renal apoptosis in MRL/lpr mice (Figure 3. G-K). Our results indicate that Res alleviates renal injury in LN mice by suppressing renal inflammation, oxidative stress, and cellular apoptosis.

### Regulatory effect of Res on Sirt1 in renal tissue of LN mice

**Figure 4:**
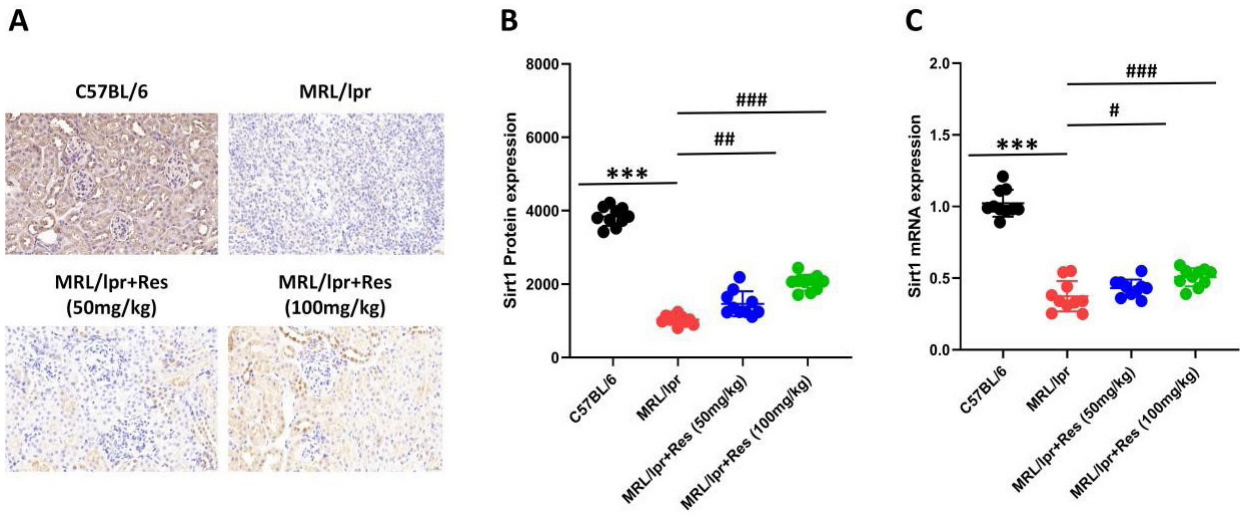
Res regulates the expression of Sirt1 protein and mRNA in the kidneys of LN mice

Previous studies have demonstrated that Sirt1 plays a pivotal role in regulating the activation of inflammatory cytokines, immune responses, and cellular apoptosis (15). Our findings revealed that Sirt1 protein was highly expressed in renal tissues of C57BL/6 mice, with predominant localization in renal tubular epithelial cells and glomerular mesangial cells (Figure 4. A). In contrast, Sirt1 protein levels were reduced to varying extents in kidney tissues of both MRL/lpr mice and MRL/lpr mice treated with Res. In contrast, significant recovery of Sirt1 protein expression was observed in MRL/lpr mice receiving 100 mg/kg Res (Figure 4. A, B). Furthermore, to validate these results, we quantified Sirt1 mRNA expression in kidney tissues using RT-qPCR. The expression pattern of Sirt1 mRNA was found to be consistent with that of Sirt1 protein (Figure 4. C). Based on these findings, we hypothesize that Res may protect renal function in LN mice by restoring Sirt1 activity.

## Discussion

LN represents one of the most serious and prevalent complications of SLE, with immune hyperactivity and renal impairment as its primary clinical manifestations (16). A majority of LN patients ultimately progress to end-stage renal disease. At present, there are no specific drugs for the treatment of LN. Hormone shock therapy and immunosuppressive therapy are mainly used in clinical practice. However, the use of these two main drugs is accompanied by various serious side effects and can aggravate kidney damage. The discovery of low-side-effect, highly effective LN treatment drugs has significant research implications. TCM usually exerts therapeutic effects on diseases through multi-target, multi-channel, and bidirectional regulation. Clinical use of TCM mainly involves animal and plant drugs, which have relatively mild medicinal properties and minimal toxic side effects. The application of TCM in the treatment of LN represents a key direction in research on LN management (17). 

Studies have found that Res exerts effects of moistening the lung, tonifying the kidney, and nourishing the blood (18). In modern pharmacology, Res has also been proven to have value in anti-inflammatory, anti-hypoxic, anti-aging, and cardiovascular and cerebrovascular protection (19, 20). The extensive medicinal value of Res has sparked interest in its potential for treating LN. Previous studies have shown that Res has great potential for treating SLE, but there are few reports on whether it can also exert therapeutic effects when SLE is secondary to nephrotic syndrome (21). Therefore, we used Res intervention in MRL/lpr mice to confirm its specific therapeutic effect and possible molecular mechanisms. 

In the present study, we employed the MRL/lpr lupus mouse model to investigate the efficacy of Res in the treatment of LN. Following 6 weeks of Res administration in MRL/lpr mice, significant improvements were observed in renal function, proteinuria, and renal histopathological changes. These findings indicate that Res treatment markedly alleviates localized renal damage in MRL/lpr mice. Further research found that spleen weight and spleen index were significantly lower than in the disease group, suggesting that Res can alleviate systemic immune activation in lupus mice. Moreover, we also found that Res could inhibit the expression of circulating inflammatory factors, oxidative stress levels, and renal cell apoptosis in lupus mice. The results of our study indicated that Res had broad application prospects in the treatment of LN. Due to the systemic bioavailability of oral Res typically ranging from 1% to 5%, we administered Res to mice at two different concentrations in the hope of exploring the optimal concentration and providing a reference for future human trials. However, Res is currently used only as an adjuvant and cannot replace core treatments such as glucocorticoids, immunosuppressants, or biologics. Res has shown therapeutic potential for LN, and it is believed to play a greater role in the future. 

Sirt1 is a histone-dependent deacetylase that relies on NAD+and can participate in regulating various biological processes, such as energy metabolism, aging, immune regulation, etc. (22-24). Currently, multiple studies have shown that Sirt1 also plays a role in immune-related diseases. In the colon of colitis model mice, Sirt1 expression was reduced, and after treatment with the Sirt1 agonist Res, acute colitis-induced inflammation was alleviated (25). Sirt1 expression increased in CD4+T cells of MRL/lpr mice, and after Sirt1 siRNA treatment, lupus pathological damage was reduced (26). In the arthritis mouse model, compared with wild-type mice, Sirt1 knockout mice showed milder joint inflammation and injury and reduced expression of inflammatory cytokines, matrix metalloproteinases, and ROR-γT (27). The research mentioned above suggests that Sirt1 may be involved in the pathogenesis and progression of LN and may exert a crucial role in the disease process.

For this purpose, we constructed an LN model using MRL/lpr mice. We observed that the expression levels of both Sirt1 protein and mRNA were significantly down-regulated in the renal tissues of MRL/lpr mice, whereas they were highly expressed in those of C57BL/6 mice. Drawing on previous studies, we hypothesize that Sirt1 exerts a protective role in the pathogenesis of LN. To verify the protective mechanism of Res, we treated MRL/lpr mice for 7 weeks. We observed a significant increase in Sirt1 expression, suggesting that Res may protect LN by activating Sirt1 to mediate immune function recovery. 

Our research has the following limitations. Firstly, the MRL/lpr mice used in this study are not Sirt1 knockout mice and lack further mechanistic research. Secondly, this study used the Sirt1 agonist Res to investigate the effects of MRL/lpr on kidney function, without reverse validation using inhibitors. The study only focused on changes in kidney function at a single time point and could not dynamically reflect systemic changes. Thirdly, this study is limited to the animal level and requires an in-depth investigation of cellular mechanisms. Clinical trials are needed to validate its efficacy and realize its full potential.

(A) SCr levels; (B) BUN levels; (C) pKIM-1 levels; (D) urinary protein levels; (E) pathological changes in renal tissue. Abbreviation: Res, resveratrol; LN, lupus nephritis; SCr, serum creatinine; BUN, blood urea nitrogen; pKIM-1, plasma kidney injury molecule-1. Vs. C57BL/6 mice, ***P<0.001; vs. MRL/lpr mice, #*P*<0.05, ##*P*<0.01, ###*P*<0.001.

(A) spleen weight; (B) spleen index; (C) thymus weight; (D) thymus index; (E) splenic dendritic cells; (F) Th1 lymphocytes; (G) Th2 lymphocytes; (H) Th1/Th2 cell ratio; (I) Treg lymphocytes. Res: Resveratrol; LN: Lupus nephritis. Vs C57BL/6 mice, **P<0.01, ***P<0.001; vs MRL/lpr mice, #*P*<0.05, ##*P*<0.01, ###*P*<0.001.

(A) TNF-α levels; (B) IL-1β levels; (C) IL-6 levels; (D) ROS levels; (E) MDA levels; (F) SOD levels; (G) apoptosis of the kidney; (H) apoptotic rate of kidney; (I) Caspase-3 mRNA levels; (J) Bax mRNA levels; (K) Bcl-2 mRNA levels. Res: Resveratrol; LN: Lupus nephritis; ROS: Reactive oxygen species; MDA: Malondialdehyde; SOD: Superoxide dismutase. Vs C57BL/6 mice, ***P<0.001; vs MRL/lpr mice, #*P*<0.05, ##*P*<0.01, ###*P*<0.001.

(A) expression of Sirt1 protein; (B) IOD values of Sirt1 protein in kideny; (C) level of Sirt1 mRNA. Res: Resveratrol; LN: Lupus nephritis; Sirt1: Sirtuin 1; IOD: Integrated optical density. Vs C57BL/6 mice, ***P<0.001; vs MRL/lpr mice, #*P*<0.05, ##*P*<0.01, ###*P*<0.001.

## Conclusion

As a TCM formulation, Res plays a significant role in the treatment of LN. It enhances renal function by stabilizing systemic immune activation in LN mice, inhibiting the expression of circulating inflammatory factors, reducing oxidative stress, and suppressing renal cell apoptosis. The specific mechanism underlying these effects is likely closely associated with the activation of the Sirt1 signaling pathway.

## Data Availability

The data used to support this study’s findings are included within the article. The datasets generated and analyzed during the current study are available from the corresponding author upon reasonable request.

## Declaration

We have not used any AI tools or technologies to prepare this manuscript.

## Animal Studies

All animal experiments in this study were performed strictly in accordance with international standard protocols and have been approved by the Experimental Animal Ethics Committee of Ningbo University (ID: AEWC-20240511).
